# Molecular Detection and Environment-Specific Diversity of Glycosyl Hydrolase Family 1 β-Glucosidase in Different Habitats

**DOI:** 10.3389/fmicb.2016.01597

**Published:** 2016-10-13

**Authors:** Rameshwar Tiwari, Kanika Kumar, Surender Singh, Lata Nain, Pratyoosh Shukla

**Affiliations:** ^1^Enzyme Technology and Protein Bioinformatics Laboratory, Department of Microbiology, Maharshi Dayanand UniversityRohtak, India; ^2^Division of Microbiology, ICAR-Indian Agricultural Research InstituteNew Delhi, India; ^3^ICAR-National Research Centre on Plant Biotechnology, LBS Centre, Indian Agricultural Research InstituteNew Delhi, India

**Keywords:** GH1 β-glucosidase, metagenomics, metabolic profiling, operational taxonomic units, microbial community

## Abstract

β-glucosidase is a crucial element of the microbial cellulose multienzyme complex since it is responsible for the regulation of the entire cellulose hydrolysis process. Therefore, the aim of the present work was to explore the diversity and distribution of glycosyl hydrolase family 1 β-glucosidase genes in three different environmental niches including, Himalayan soil, cow dung and compost by metagenomic approach. Preliminary evaluation through metabolic profiling using BIOLOG based utilization patterns of carbon, nitrogen, phosphorus and sulfur revealed the environment and substrate specific nature of the indigenous microbial population. Furthermore, clonal library selection, screening and sequence analysis revealed that most of the GH1 β-glucosidase proteins had low identities with the available database. Analysis of the distribution of GH1 β-glucosidase gene fragments and β-glucosidase producing microbial community revealed the environment specific nature. The OTUs obtained from Himalayan soil and compost metagenomic libraries were grouped into 19 different genera comprising 6 groups. The cow dung sample displayed the least diversity of GH1 β-glucosidase sequences, with only 14 genera, distributed among three groups- Bacteroidetes, Firmicutes, and Actinobacteria. The metagenomic study coupled with metabolic profiling of GH1 β-glucosidase illustrated the existence of intricate relationship between the geochemical environmental factors and inherent microbial community.

## Introduction

Cellulose is the most abundant fixed form of carbon (100 billion ton global production per year) in the biosphere. This can be converted for sustainable energy production through its sequential bioconversion (Dusselier et al., 2014; Brown, 2015). Cellulose microfibrils contain, closely packed crystalline two to three dozen (1 → 4)-β-D-glucan chains and disordered amorphous regions that are randomly distributed along with their length (Percival Zhang et al., 2006). The complex native cellulose architecture, explains the evolutionary development in microbial cellulose degradation mechanism (Béguin, 1990). Cellulose hydrolysis can be performed under polysaccharide-enzyme synergy, even in the absence of microbial cells. The complete enzymatic hydrolysis of cellulose is accomplished by a set of cellulase enzymes belonging to the Glycosyl Hydrolase (GH) family (EC: 3.2.1) (Davies and Henrissat, 1995; Franková and Fry, 2013; Elleuche et al., 2014). These enzymes hydrolyze the glycosidic bond between various carbohydrates or between a carbohydrate and a non-carbohydrate moiety. Typically, enzymatic hydrolysis of cellulose requires three different types of cellulase enzymes, namely, endoglucanase (EC 3.2.1.4), exoglucanase (EC 3.2.1.91) and β-glucosidase (EC 3.2.1.21) (Bayer et al., 1998; Percival Zhang et al., 2006). The endoglucanase degrades cellulose to shorter cellooligosaccharides; while the exoglucanase cleaves the reducing and/or non-reducing ends of the cellulose moiety to produce cellobiose, which are finally hydrolyzed by β-glucosidase into glucose. β-glucosidase is a key rate limiting factor of the cellulose complex, since it hydrolyzes the end dimer “cellobiose” (Cairns and Esen, 2010). This enzyme is responsible for the regulation of the entire cellulose hydrolysis process by easing cellobiose-mediated suppression and producing the final product glucose (Singhania et al., 2013). This enzyme is not only involved in hydrolyzing the glycosyl bond, but also reverse of hydrolysis which is catalyzed under specific conditions (Bhatia et al., 2002). The glycosyl hydrolase family β-glucosidase is grouped into GH1 and GH3 families (http://www.cazy.org/) (Cantarel et al., 2009). Among them, the GH1 family proteins exhibit β-glucosidases, β-galactosidases or proteins with both activities, having considerably higher Km values for the galactosides (Cairns and Esen, 2010). At present, a total of 329 GH1 family β-glucosidases have already been characterized from bacteria, archaea and eukaryotes. The protein database also comprises 47 crystal structures of GH1 family proteins, including those of several microbial β-glucosidases, β-galactosidases, β-rutinosidases, alkyl β-glucosidases, 6-P-β-glucosidases and 6-P-β-galactosidases (Aguilar et al., 1997; Wierzbicka-Woś et al., 2013). Moreover, GH1 family proteins possess a single active domain with an α/β barrel topology, while GH3 proteins are made up of two domains. GH1 β-glucosidases are more active than GH3 on di- or oligosaccharides (Cota et al., 2015) and also tolerate end product (glucose) upto a high limit (Giuseppe et al., 2014). Due to these properties, GH1 β-glucosidases are being recognized as versatile biocatalysts, with numerous applications in biorefinery, industry, medicine and food. Therefore, in view of its versatility and utility, it is very important to decipher GH1 β-glucosidase producing microbial communities from diverse environmental niches.

Previously, various microorganisms were evaluated on the basis of their β-glucosidase production potential (Tiwari et al., 2013, 2014, 2015). However, current cultural laboratory practices are unable to illustrate the microbial diversity due to spatial heterogeneity, non-ubiquitous nutrient requirements and taxonomic ambiguity among the microbial population in the environment. Also, few studies on ecological screening, process optimization and enzymatic applications in industries are broadening the scope and new niche for their utilization (Shukla et al., 2007; Zhang et al., 2010; Banerjee et al., 2016; Baweja et al., 2016). Additionally, the modern techniques of gene editing, systems biology, molecular modeling of enzyme are the notable areas, where the innovation can provide novel products and applications (Singh and Shukla, 2011; Karthik and Shukla, 2012; Baweja et al., 2015; Gupta and Shukla, 2016b; Kumar et al., 2016; Singh et al., 2016).

Metagenomics offers an opportunity to explore both microbial diversity and mine novel biocatalysts from environmental DNA (Ferrer et al., 2005; Wang et al., 2009; Vey and Moreno-Hagelsieb, 2012). This approach has been used successfully to identify enzymes with defined activities (Kim et al., 2008; Wang et al., 2009), it initially depends on relatively low-throughput function-based screening of environmental DNA clone libraries (Simon et al., 2009; Uchiyama and Miyazaki, 2009; Gupta and Shukla, 2016a). Most of the research work focuses on microbial ecology based taxonomic divergence which contributes a lot toward understanding the richness of microbial populations in specific environmental niche (Achtman and Wagner, 2008; Haegeman et al., 2013). Although, few attempts have been made to explore the microbial diversity on the basis of their metabolic or functional potential (Liang et al., 2011; Engel et al., 2012), metagenomic approach may facilitate mimicking the biological processes in the specific environmental condition in a more meaningful manner (Baweja et al., 2015). Moreover, the functional diversity analysis of a biocatalyst through metagenomic approach also defines the hidden majority of uncultured microbes(Arnosti, 2011; Kumar Singh and Shukla, 2015). Many β-glucosidase genes have been extracted from various environmental niches like alkaline polluted soil (Wang et al., 2012), gut microbiome (Del Pozo et al., 2012), hot spring (Schröder et al., 2014), agricultural soil (Biver et al., 2014), marine water (Fang et al., 2010), mangrove soil (Li et al., 2012), compost (McAndrew et al., 2013) and anaerobic digester (Wang et al., 2015) through unculturable metagenomic approaches but only a few of their functional proteins have been characterized in detail (Fang et al., 2010; Li et al., 2012; Wang et al., 2012). Therefore, metagenomic based mining of β-glucosidase genes, in order to explore the similarities and differences can strengthen our knowledge about the functional diversity of β-glucosidase from different environmental niches. Congregated compilation of this genetic information across different environmental niches, including extreme conditions, of GH1 β-glucosidase, using metagenomic approach will be vital for improving the prospects of improving cellulosic bioethanol production.

## Materials and methods

### Sample collection for phenotypic and metagenomic analysis

The cold desert of southeastern Himalayan region, Kargil district (KD), India (34° 32′ North latitude and 76° 07′ East longitude) was selected for soil sample collection at high altitude (10,500 ft above sea level). The annual temperature varies from −20 to 5°C. Compost (CM) was prepared from paddy straw collected from the premises of Division of Microbiology, IARI, New Delhi, India. At the time of collection, compost heap was in the thermophilic phase with temperature ranging between 60° and 65°C. Cow dung (CD) was collected from dairy farm situated in Todapour village, New Delhi, India. All samples were kept at 4°C during transportation and therafter at −20°C in the laboratory.

### Community-level substrate utilization by different environmental samples by BIOLOG phenotypic microarray

The soil samples from Kargil District (KD), Compost (CM), and Cow dung (CD) were analyzed based on utilization of carbon, nitrogen, phosphorus and sulfur substrate using the BIOLOG phenotypic microarray PM MicroPlates (Biolog, Inc., Hayward, California, USA). Phenotype MicroPlates PM1-2, PM3B and PM4A measure the carbon, nitrogen and phosphorus/sulfur metabolism, respectively. The samples were suspended in sterile saline water (0.8% w/v) and incubated under shaking condition (100 rpm) for 3 h at room temperature for proper mixing. The mixture was allowed to settle and aliquots were diluted in 15 ml of inoculation fluid (IF-0; Biolog) to adjust the optical density at 420 nm of 0.2 which were then used as the inoculum for the Microplates (Weber et al., 2007). For PM3B and PM4A plates, 15 ml inoculation fluid was added to the mixture of sodium succinate and ferric citrate as carbon sources. The 100 X additive solutions were prepared by dissolving 5.402 g of sodium succinate and 0.49 mg ferric citrate in sterile saline water. A total of 150 μl from the 100 X solution were added to the 15 ml of IF-0 inoculating fluid before preparing the sample suspensions. Thereafter, 100 μl of sample suspension was inoculated into each PM well. All PM plates were used in triplicate for each sample and were incubated at 30°C. After 72 h of incubation, each well was inoculated with 100 μl of the Biolog redox dye mix. For color development, the plates were incubated at 37°C until violet color appeared. Absorbance readings were taken at 590 nm with a plate reader (Micro Plate. Reader) (Weber et al., 2007).

### Data normalization and analysis

The absorbance data were normalized to observe the average good color development (AWCD) as mentioned by (Garland, 1999) and further selected for analysis.

The normalized absorbance for each well was calculated as:

$$\text{AWCD}=\Sigma {\text{OD}}_{\text{i}}/95$$

Where OD_i_ is the absorbance value from each well, corrected subtracting the blank well (A1) values from each plate well and 95 is the number of total substrates.

Substrate richness (S) in selected sample was calculated as the number of wells with a corrected absorbance greater than 0.25 (Siragusa et al., 2009).

The Shannon index (H) or “diversity” was calculated to understand the common ecological metric and the microbial communities shift over space and time. The Shannon–Weaver index was calculated as follows:

$$\text{H}=-\Sigma {\text{p}}_{\text{i}}ln(\text{pi})$$

Where *p*_*i*_ is the ratio of the activity on each substrate (OD_i_) to the sum of activities on all substrates ΣOD_i_.

The phenotypic expression level and *t*-test based significance was considered by using Multiple array viewer (MeV 4.9.0) software.

### Degenerate primer designing for β-glucosidase gene amplification

A total of 247 glycosyl hydrolase Family 1 β-glucosidase amino acid sequences were retrieved from NCBI Genbank. Protein sequences were aligned using PROMALS3D (http://prodata.swmed.edu/promals3d/promals3d.php) to predict conserved regions and secondary structure based on available homologous 3D structures in database. All sequences were edited and trimmed to remove short, ambiguously aligned regions. The trimmed alignment was analyzed and graphical representation of conserved region was generated through weblogo3 interface (http://weblogo.threeplusone.com/create.cgi) (Crooks et al., 2004). Based on lowest degeneracy, equal GC content and Tm value, the following primers were designed: BGLTF (27): 5′ ACI YTI TAY CAY TGG GAY YTI CCI CAR 3′; BGLTR (27): 5′ RTA ICC YTC IGC CCA YTC RAA RTT RTC 3′**;** BGL21F (21): 5′ TAY CAY TGG GAY YTI CCI CAR 3′**;** BGL21R (21): 5′ RTA ICC YTC IGC CCA YTC RAA 3′**;** BGL17F (21): 5′ GCG CCT AYC AYT GGG AYY TIC C 3′**;** BGL17R (17): 5′ CCY TCI GCC CAY TCR AA 3′. Inosine (I) was used to decrease the degeneracy. The efficiency of these primer sets was confirmed by *in silico* PCR through a NCBI primer BLAST search with different microorganism name, microorganism group or taxonomy ID.

### Extraction of metagenomic DNA from samples

DNA extraction from soil sample of Kargil District (KD), Compost (CM), and Cow dung (CD) was performed using the Power Soil DNA Isolation Kit (MoBio Laboratories Inc., Carlsbad, USA) as per the manufacturer's instruction.

### Amplification of partial β-glucosidase gene

PCR amplification was performed using high fidelity *Pfu* DNA polymerase (Fermentas) with proofreading activity to avoid Taq polymerase based low replication fidelity. Reactions were carried out in a BioRed PCR system with metagenomic DNA as template. PCR condition was optimized for all parameters of PCR by gradient PCR (50–58°C), different MgCl_2_ concentration (1–2.5 mM) and primer concentration (0.1–0.4 mM). The final PCR was conducted by using primers sets forward primer BGL17F (5′ GCG CCT AYC AYT GGG AYY TCC 3′) and reverse primer BGLTR (5′ RTA CCY TCG CCC AYT CRA ART TRT C 3′). The reaction mixture contained: 10 μl: 10X Pfu DNA polymerase buffer with MgCl_2_, 0.3 mM: dNTP mix (10 mM), 0.1 μM each: Primers, 2 units: Pfu DNA polymerase, 10–50 ng: metagenomic DNA and MQ water make the final volume up to 100 μl. The PCR program was as follows: initial incubation at 95°C for 5 min, followed by 40 cycles (95°C for 50 s, 55°C for 1 min, and 72°C for 90 s), and then by a final extension at 72°C for 10 min. The amplified products were analyzed by electrophoresis at 60 V for 1 h in 1.2% agarose gel in 1X TAE buffer.

### Construction of metagenomic library in pGEM-T easy vector

The pGEM-T Easy vector (Promega) was used to construct the three metagenomic libraries of partial β-glucosidase gene. Since, *Pfu* DNA polymerase is unable to add “A” overhang, “A” tail was generated in the PCR product. The PCR product was purified using the Qiaquick PCR purification kit (Qiagen, Valencia, CA, USA) as per manufacture's instruction. The purified PCR products of three environmental samples were first cloned in pGEM-T Easy vector. The pGEM-T and β-glucosidase construct was transformed into competent cells of *E. coli* strain (DH5α) and colonies were screened by blue/white colony selection on the LB plates containing X-gal (80 μg/ml), IPTG (0.5 mM) and ampicillin (100 mg/ml).

### Plasmid isolation and amplification of partial β-glucosidase genes

All positive white colonies were further inoculated to the 5 ml LB broth for plasmid isolation. QIAprep Spin Miniprep Kit (Qiagen, Valencia, CA, USA) was used for plasmid extraction as per manufacture's instruction. Clones containing putative β-glucosidase genes were screened by amplification of the isolated plasmids with M13F and M13R primers.

### Screening for clonal duplication

In order to avoid sequencing several identical β-glucosidase genes, Restriction Fragment Length Polymorphism (RFLP) analysis was performed by treating the PCR products (amplified with M13 primers) with four base pair-cleaving restriction endonuclease *Alu*I (MBI Fermentas) at 37°C for 12 h, and the resulting electrophoretic mobility at 3% agarose was used to group the isolates.

### Sequencing and analysis

The PCR products were sequenced by the Sanger sequencing method at Xcelris Labs Ltd, Ahmedabad, India. The obtained sequences were checked for chimera and homology were analyzed by the BLASTx search in the National Center for Biotechnology Information (NCBI) database (http://www.ncbi.nlm.nih.gov) and were submitted to NCBI Genbank. The nucleotide sequence was further translated into amino acid sequence (http://web.expasy.org/translate). All sequences of the same environmental niches were aligned using Clustal W version 1.8. The evolutionary distances were calculated and phylogenetic dendrogram was constructed by neighbor-joining method and tree topologies were evaluated by performing bootstrap analysis of 1000 data sets using MEGA 5.0 (Molecular Evolutionary Genetics Analysis).

## Results

### Phenotypic microarray based substrate utilization pattern

**Figure 1:**
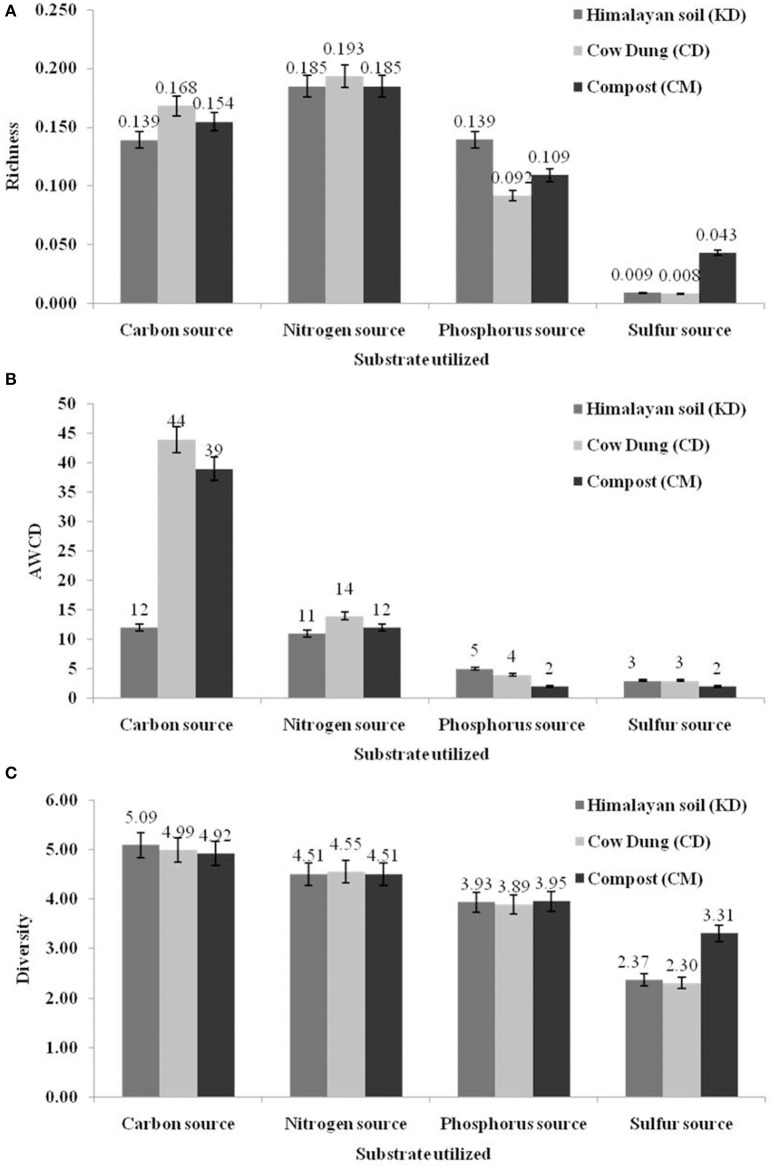
**Diversity indices based on community level physiological profiling (CLPP) *p*-values based on *t*-tests**. **(A)** Richness (R); **(B)** Average well-color development (AWCD) and **(C)** Shannon–Weaver index (H) of metabolized substrates.

The metabolic profiling of the indigenous microbial population of three samples from the Himalayan region, Kargil district (KD), India, Cow dung (CD) and Compost (CM) was analyzed by BIOLOG based substrate carbon (*n* = 190), nitrogen (*n* = 95), phosphorus (*n* = 59) and sulfur (*n* = 35) utilization pattern. Figure 1 shows clear differences among the substrate utilization patterns in microbial communities of diverse environmental samples, as evident from AWCD, richness, and diversity values. The *P* values were ≤ 0.05 which showed the significance of the experiment. For carbon and nitrogen sources, all the three AWCD and richness values were greater for Cow dung (CD), followed by CM and KD.

The normalized data were further utilized for hierarchal clustering to explore the possible linkages between substrate utilization patterns and three different samples. On the basis of 1.0 distance threshold, all 190 carbon sources were grouped into 5 major clusters (Supplementary Figure 1). Himalayan soil and cow dung sample were grouped closely which showed a common carbon utilization pattern. The clustering of nitrogen sources showed a very diverse utilization pattern, as only one major cluster was formed. The phosphorus and sulfur substrates were grouped into 3 clusters. According to the *t*-test based, on –log_10_ (*p*) values, 86% (*n* = 164) carbon sources were utilized by the microbial community of all three samples, while the rest of the 14% (*n* = 26) registered non–significant utilization. Similarly, for nitrogen, phosphorus and sulfur substrates, 97, 90, and 86% substrates were utilized significantly by all the samples, respectively.

### Sequence analysis of GH1 family β-glucosidase for primer designing

**Figure 2:**
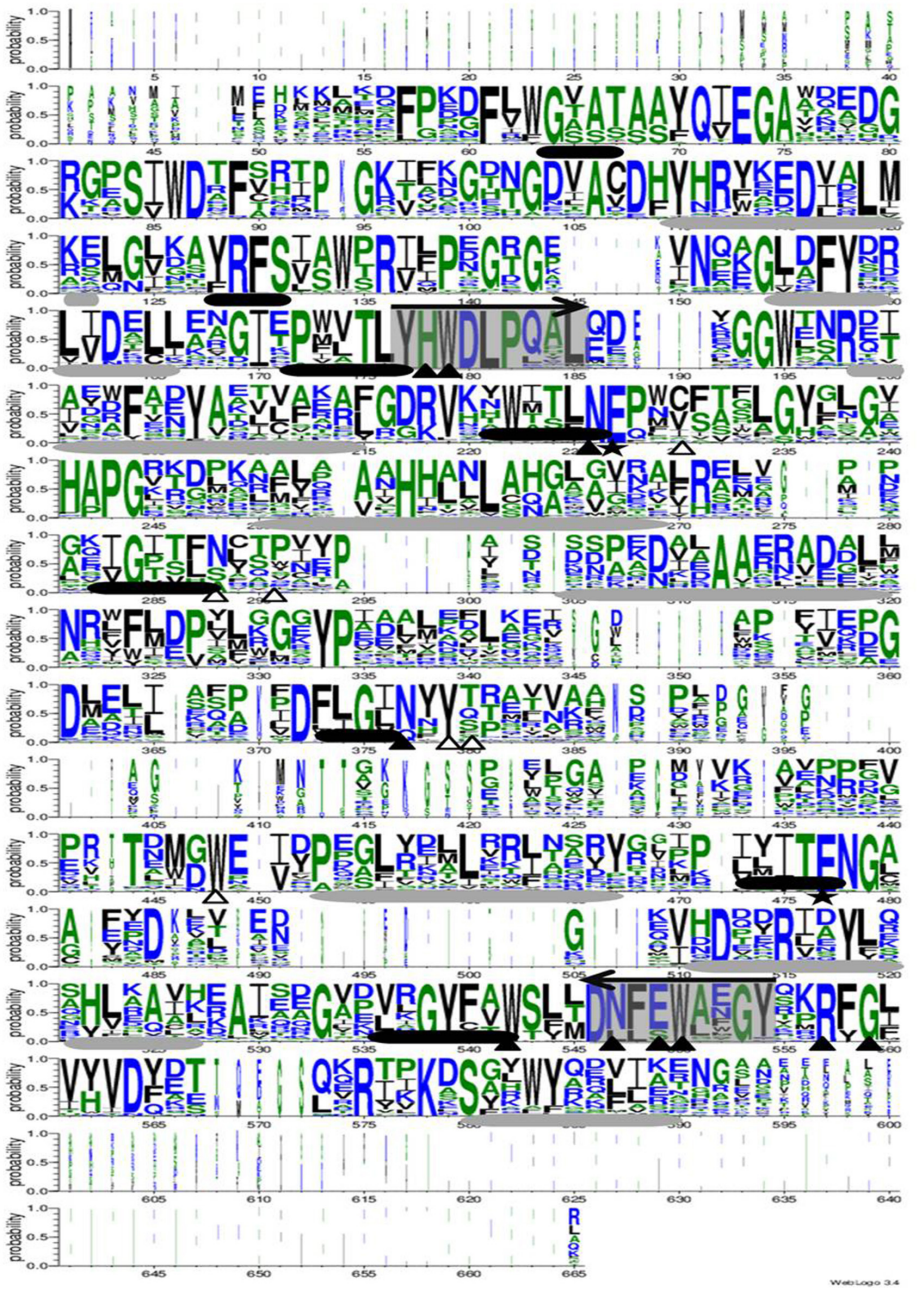
**The sequence logo of 247 glycosyl hydrolase Family 1 β-glucosidase amino acid sequences based on alignment using PROMALS3D**. The identical and conservative residues are shown in *white and black triangle*, respectively. The two catalytic glutamate residues are shown by *stars*. The black and gray cylinders shown β and α sheets, respectively. The arrow represented the selected conserved region for degenerate primer design.

The alignment of 247 glycosyl hydrolase Family 1 β-glucosidase amino acid sequences was analyzed and graphical representation of conserved region was generated (Figure 2). The alignment showed characteristic features of GH1 with secondary structure of a classical (α/β)_8_ sheets. The presence of two catalytic glutamate residues being 200 residues apart from each other and located at the C-terminal ends of β-strands 4 (acid/base) and 7 (nucleophile). Other characteristic identical and conserved residues are also shown in Figure 2. The peptide sequences [**T**/C]-[**L**/I/V/M/A]-[**Y**/F]-**H**-**W**-**D**-**L**-**P**-**Q** and **D**-[**N**/L]-[**F**/L]-[**E**/S]-**W**-[**A**/L/G/V/S]-[**E**/N/F/W/M/L]-**G**-[**Y**/I/E/L/F] were found to be the most conserved throughout the consensus sequence according to the maximum length of the peptide. Based on the maximum probability peptide sequence TLYHWDLPQ and DNFEWAEGY were selected to design forward and reverse degenerate primers, respectively. The selected region was pretended to amplify approx. 876 bp of PCR product.

### Amplification of partial β-glucosidase gene

The metagenomic DNA was extracted from all three samples and used as a template for amplifying the partial β-glucosidase gene. The synthesized primer sets were used to optimize the PCR condition for amplification of PCR products with the desired length. Gene fragments, about 876 bp for β-glucosidase gene, were amplified from the primer pair BGL17F/BGLTR (Supplementary Figure 2).

### Construction of metagenomic library in pGEM-T easy vector

The purified PCR products of three environmental samples were separately cloned in pGEM-T Easy vector for metagenomic library construction. The positive clones were selected based on blue/white colony screening. After random confirmation of 80 clones from each library by PCR with primers M13F and M13R, a total of 60 clones was sequenced from three metagenomic libraries.

### Restriction fragment length polymorphism (RFLP) analysis of PCR products amplified from selected clones

The identical clones were selected by Restriction Fragment Length Polymorphism (RFLP) analysis of 90 PCR products obtained by M13 primers from selected clones by restriction endonuclease *Alu*I (Supplementary Figure 3). On the basis of restriction pattern, 20 clones were selected from each metagenomic library for further sequencing.

### Sequencing and phylogenetic analysis of partial β-glucosidase (BGL) gene

**Figure 3:**
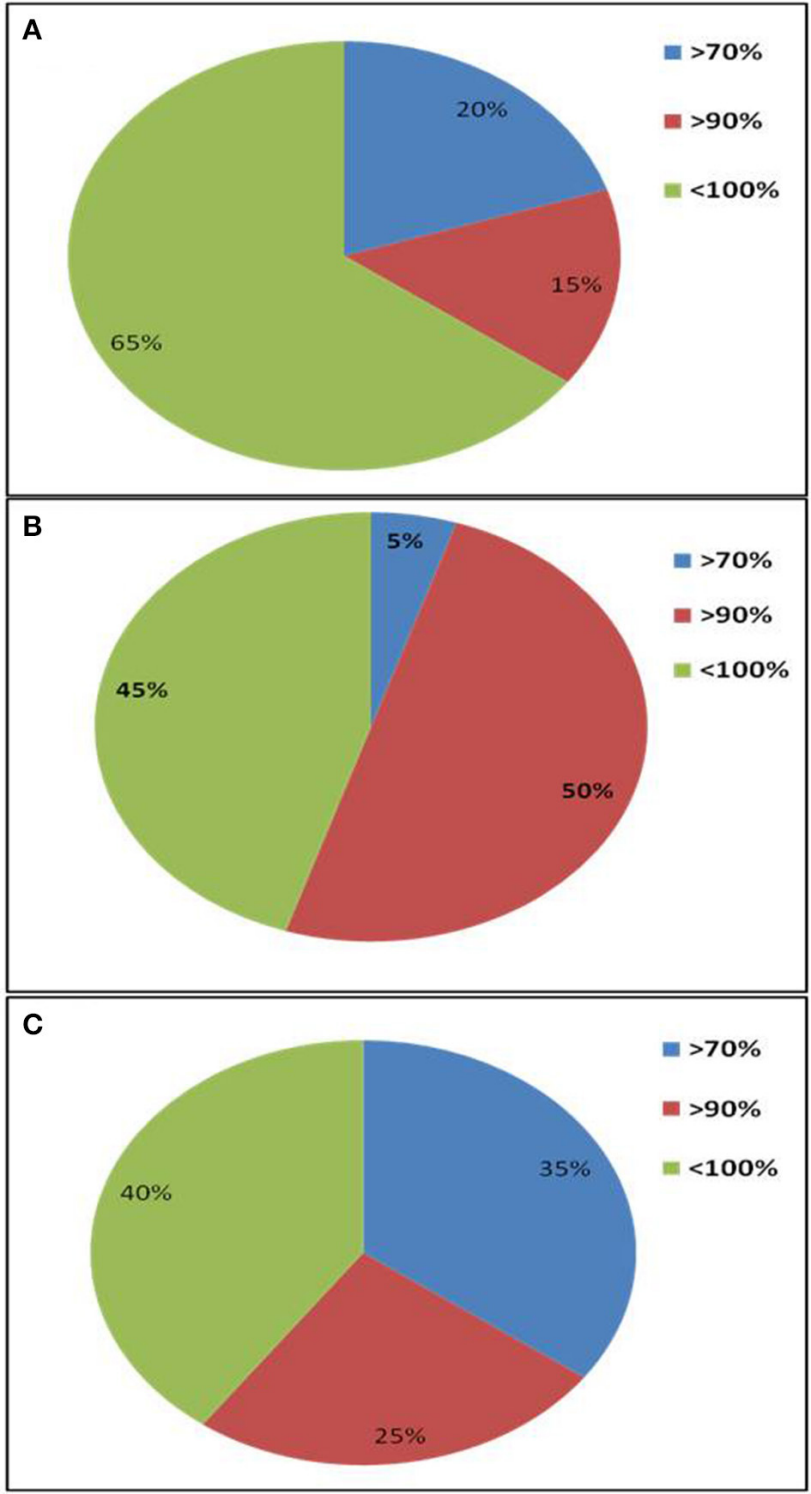
**Amino acid sequence identities of GH1 β-glucosidase gene fragments from three environmental niches based on NCBI-BLASTp search**. **(A)** Himalayan soil (KD); **(B)** Cow dung (CD); **(C)** Compost (CM).

A total of 20 sequences of β-glucosidase genes were obtained from each metagenomic library. All sequences were identified as glycosyl hydrolase family 1 β-glucosidase (BGL) gene by BLASTx search and contained the two catalytic glutamic acid residues. The sequences were submitted to NCBI GenBank under accession numbers, KT898053- KT898072 for compost (CM), KT898073- KT898092 for cow dung (CD) and KT898093- KT898112 for Himalayan soil (KD) (Table 1). Based on the NCBI-BLASTp analysis, 20, 5, and 35% sequences had low identities (< 70%) with known GH1 family β-glucosidase sequences in GenBank from the Himalayan soil, cow dung and compost sample, respectively (Figure 3).

**Table 1:** **Sequencing details of selected clones from each metagenomic library based on their deduced amino acid sequence identities**.

**S. No**.	**Clone ID**	**Nearest neighbor**	**Division**	**Identity (%)**
**HIMALAYAN SOIL (KD)**				
1	KD1	*Vibrio vulnificus*	γ-Proteobacteria	60
2	KD2	*Meiothermus ruber*	Deinococcus	59
3	KD3	*Arcticibacter svalbardensis*	Bacteroidetes	83
4	KD4	*Olivibacter sitiensis*	Bacteroidetes	69
5	KD5	*Segetibacter koreensis*	Bacteroidetes	69
6	KD6	*Flavobacterium sp.*	Bacteroidetes	76
7	KD7	*Exiguobacterium sp.*	Firmicutes	99
8	KD8	*Tumebacillus flagellatus*	Firmicutes	98
9	KD9	*Alteromonas macleodii*	γ-Proteobacteria	98
10	KD10	*Ensifer adhaerens*	α-Proteobacteria	96
11	KD11	*Azospirillum brasilense*	α-Proteobacteria	99
12	KD12	*Rhodopseudomonas palustris*	α-Proteobacteria	97
13	KD13	*Rhodovulum sp.*	α-Proteobacteria	98
14	KD14	*Burkholderia mimosarum*	β-Proteobacteria	98
15	KD15	*Halobacillus sp*	Firmicutes	97
16	KD16	*Thermobacillus composti*	Firmicutes	98
17	KD17	*Thermoanaerobacterium xylanolyticum*	Firmicutes	98
18	KD18	*Paenibacillus sp.*	Firmicutes	98
19	KD19	*Devosia limi*	α-Proteobacteria	87
20	KD20	*Sphingobacterium sp.*	Bacteroidetes	99
**COW DUNG (CD)**				
1	CD1	*Formosa agariphila*	Bacteroidetes	89
2	CD2	*Arenibacter algicola*	Bacteroidetes	73
3	CD3	*Streptosporangium roseum*	Actinobacteria	71
4	CD4	*Paenibacillus*	Firmicutes	98
5	CD5	*Pontibacter korlensis*	Bacteroidetes	98
6	CD6	*Spirosoma radiotolerans*	Bacteroidetes	98
7	CD7	*Hymenobacter swuensis*	Bacteroidetes	97
8	CD8	*Streptomyces sp.*	Actinobacteria	95
9	CD9	*Flavobacterium sp.*	Bacteroidetes	74
10	CD10	*Flavobacterium subsaxonicum*	Bacteroidetes	74
11	CD11	*Flavobacterium sp.*	Bacteroidetes	77
12	CD12	*Flavobacterium hibernum*	Bacteroidetes	74
13	CD13	*Sphingobacterium sp.*	Bacteroidetes	84
14	CD14	*Cytophaga hutchinsonii*	Bacteroidetes	60
15	CD15	*Pedobacter sp.*	Bacteroidetes	84
16	CD16	*Pedobacter heparinus*	Bacteroidetes	83
17	CD17	*Cellulophaga baltica*	Bacteroidetes	99
18	CD18	*Clostridium] termitidis*	Firmicutes	98
19	CD19	*Clostridium straminisolvens*	Firmicutes	97
20	CD20	*Clostridium cellulovorans*	Firmicutes	97
**COMPOST (CM)**				
1	CM1	*Arenibacter algicola*	Bacteroidetes	73
2	CM2	*Verrucomicrobiae bacterium*	Chlamydiae	64
3	CM3	*Zunongwangia profunda*	Bacteroidetes	63
4	CM4	*Pontibacter korlensis*	Bacteroidetes	66
5	CM5	*Cellulophaga baltica*	Bacteroidetes	74
6	CM6	*Mucilaginibacter paludis*	Bacteroidetes	70
7	CM7	*Maribacter sp.*	Bacteroidetes	72
8	CM8	*Spirosoma lingual*	Bacteroidetes	68
9	CM9	*Adhaeribacter aquaticus*	Bacteroidetes	69
10	CM10	*Elizabethkingia miricola*	Bacteroidetes	73
11	CM11	*Vibrio vulnificus*	γ-Proteobacteria	99
12	CM12	*Gynuella sunshinyii*	γ-Proteobacteria	100
13	CM13	*Vibrio campbellii*	γ-Proteobacteria	59
14	CM14	*Devosia*	α-Proteobacteria	89
15	CM15	*Sinorhizobium fredii*	α-Proteobacteria	99
16	CM16	*Paenibacillus*	Firmicutes	99
17	CM17	*Bacillus akibai*	Firmicutes	98
18	CM18	*Actinospica acidiphila*	Actinobacteria	97
19	CM19	*Streptomyces sp.*	Actinobacteria	96
20	CM20	*Clostridium cellulovorans*	Firmicutes	98

**Figure 4:**
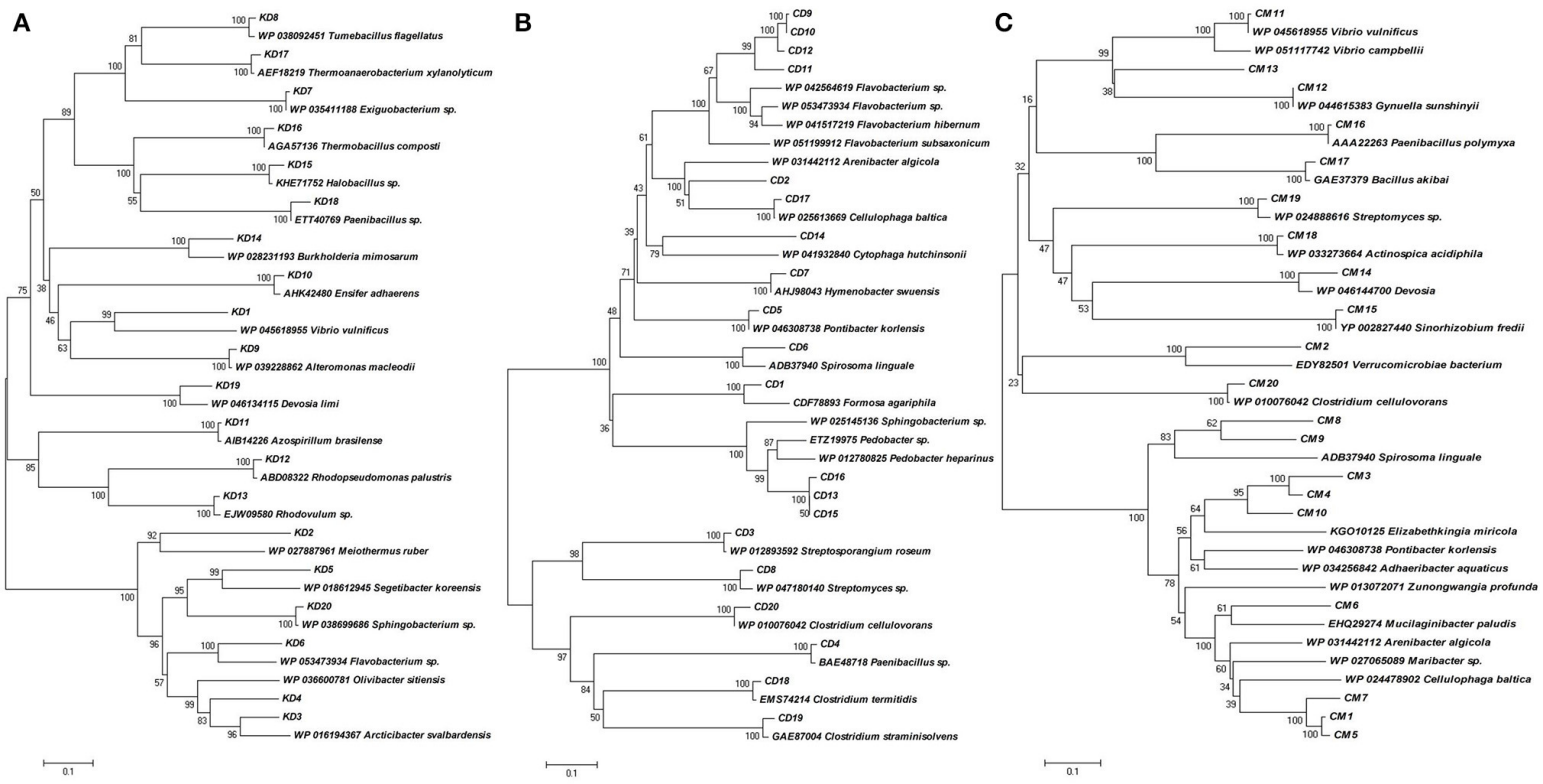
**Phylogenetic analysis based on the partial amino acid sequences of GH1 β-glucosidase genes detected from the three metagenomic libraries (A) Himalayan soil, (B) Cow dung and (C) Compost samples and their relationship with the reference sequences retrieved from GenBank**. The tree was constructed using the neighbor-joining method.

**Figure 5:**
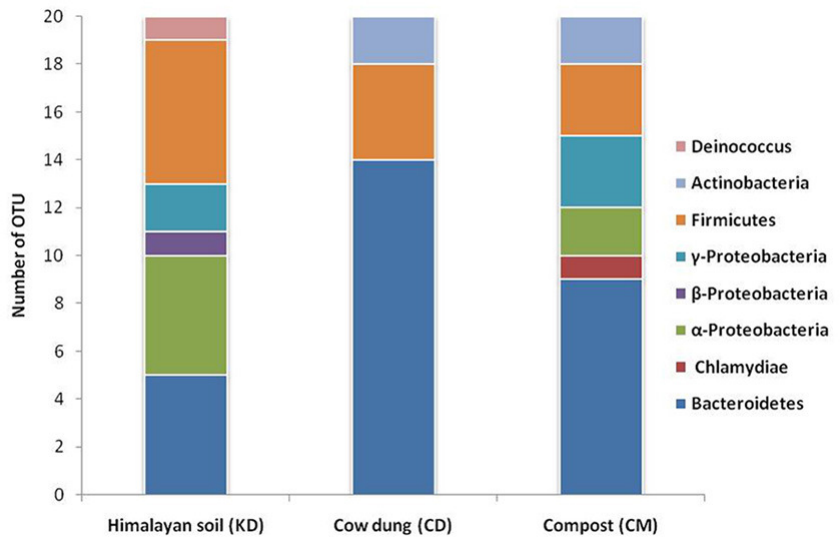
**Diversity of GH1 β-glucosidase genes based on the identified bacterial groups**.

The phylogenetic diversity of deduced protein sequences was analyzed by building the unrooted protein-level phylogenetic tree for BGL sequence OTUs (Operational Taxonomic Units) using the 20 divergent sequences from each clone library with their respective neighbor sequences retrieved from NCBI GenBank (Figure 4). Metagenomic sequences from Himalayan soil (KD) showed similarities with 19 belonging to 6 groups, including *Deinococcus*, α-Proteobacteria, β-Proteobacteria, γ-Proteobacteria, the Bacteroidetes and Firmicutes. The Compost (CM) OTUs also showed 19 genera comprising 6 groups, including Chlamydia, Bacteroidetes, α-Proteobacteria, γ-Proteobacteria, Firmicutes and Actinobacteria. The Cow dung (CD) sample showed least diversity which arranged into 14 genera with only three groups - Bacteroidetes, Firmicutes and Actinobacteria (Figure 5).

## Discussion

### Phenotypic microarray based substrate utilization pattern

The microbial metabolic activities of any particular environmental system depend on the utilization of specific nutrients. Hence, the functional diversity of environmental niches can be compared through the analyses of substrate utilization profiles (Torsvik and Øvreås, 2002). Phenotypic microarray provided by the BIOLOG may be the best way to compare the utilization of diverse group of essential substrates including carbon, nitrogen, phosphorus and sulfur (Stefanowicz, 2006). Specific substrate utilization patterns were generated by the microbial communities of three different environmental samples. The AWCD values showed that among all three samples, Himalayan soil showed least active utilization of substrates, as compared with two other samples. Cow dung and compost represent microbially induced active environmental systems as compared to the cold desert Himalayan soil. However, the diversity indices for carbon utilization was highest for the microbial community samples from the Himalayan soil, illustrating that microbial abundance is high, but not metabolically active. Carbon was utilized more efficiently by all the three environmental systems, as compared with other substrates. It is already reported that the microbial ecosystem is mainly governed based by the carbon cycle (Paerl and Pinckney, 1996). The carbon, phosphorus and sulfur substrates were grouped into 5, 3, and 3 clusters based on their combined utilization by all three samples (Supplementary Figure 1). Interestingly, nitrogen sources were utilized diversely by the microbial community and no visible clustering was observed even at 1.0 distance threshold. Nitrogen mineralization or transformation is carried out by different groups of microorganisms in environmental systems, which maybe responsible for lack of discernible groups in the different samples (Francis et al., 2007). This further ilustrates that nitrogen may not be a selective factor for distinguishing among the microbial populations from the three samples, although carbon based utilization pattern revealed that all three environments share selective and common metabolic profiles.

### Diversity and distribution of glycosyl hydrolase 1 family β-glucosidase gene

Potential producers of β-glucosidase were identified by the conventional culture based method, during previous investigations (Tiwari et al., 2014, 2015). Although, the cultural diversity is more relevant due to its applications, the unseen potential of β-glucosidase gene embedded in uncultured microbial population of diverse environmental niches needs exploration. Therefore, the GH1 family β-glucosidase protein sequences were searched extensively and based on the most conserved region, degenerate primers were designed. Although, the putative amplified region is not representative of the full length gene sequence, it encodes the sequence belongiong to the complete conserved domain sequence of the BglB superfamily.

The primer pair was used successfully to amplify the PCR product of desired size (approx. 876 bp) from all three environmental systems. Furthermore, the metagenomic library was constructed to perform the diversity and distribution studies. After the selection of positive clones, RFLP based screening and sequencing, twenty OTUs (Operational Taxonomic Units) from each metagenomic library were selected for diversity studies. Himalayan soil (KD), compost (CM) and cow dung (CD) was having 6, 11, and 10 novel OTUs based on the identities of ≤ 75% with known GH1 β-glucosidase protein sequences. The low identities implying that these β-glucosidases may be as yet undiscovered. In all three environmental niches, GH1 β-glucosidase was distributed in Bacteroidetes, Deinococcus, α, β, and γ-Proteobacteria, Firmicutes, Actinobacteria and Chlamydiae groups belonging to the total of 39 different genera. This result validated the potential of the designed degenerate primers and confirmed that primers can work over a broad range of GH1 β-glucosidases and facilitate future metagenomic studies of diverse ecological niches.

The highest diversity index and number of microbial groups were detected in the Himalayan soil sample. Himalayan samples were obtained from a cold desert where no microbial transformation was possible due to the extreme cold climate conditions; this was supported by the results of the phenotypic microarray experiment revealing lesser metabolic activity as compared with other samples. But, geographically the environment contained a hidden organic pool, as recently evidenced by Hood and co-workers (Hood et al., 2015). They informed that the mountain glaciers are storing high organic carbon content and releasing the carbon into the environment due to microbial transformation, as and when the ice shield melts due to the climate change. This concluded that cold desert environment is having low active, but high abundant microbial population.

The lowest diversity was displayed by cow dung samples, where only three groups, including Bacteroidetes, Firmicutes and Actinobacteria were present. The selective pressure on microbial load while passing through the animal gut may be the possible reason for this low diversity (Callaway et al., 2010).

## Conclusion

In conclusion, diverse GH1 β-glucosidase genes were identified from three different environmental niches using metagenomic approach. The low identities of deduced protein sequences revealed the abundance of unexplored diverse and novel β-glucosidase genes in the environment. These results provide the evidence that the distribution and diversity of β-glucosidase are habitat specific and influenced significantly by the availability of nutrients, particularly carbon, as illustrated by the metabolic profiling of abundant microbial populations.

## Author contributions

RT and LN designed the study and RT carried out all the experiment. SS and KK helped in analyzing the data. PS and LN critically written and revised the manuscript for important intellectual content. All authors read and approved the final manuscript.

### Conflict of interest statement

The authors declare that the research was conducted in the absence of any commercial or financial relationships that could be construed as a potential conflict of interest.
