# Supplementary material for: Molecular Detection and Environment-Specific Diversity of Glycosyl Hydrolase Family 1 β-Glucosidase in Different Habitats
Source: Front Microbiol. 2016 Oct 13;7:1597. doi: 10.3389/fmicb.2016.01597 (PMC5062022; doi:10.3389/fmicb.2016.01597)
Supplement: Supplementary file 1 [file DataSheet1.DOCX]

**[A]**


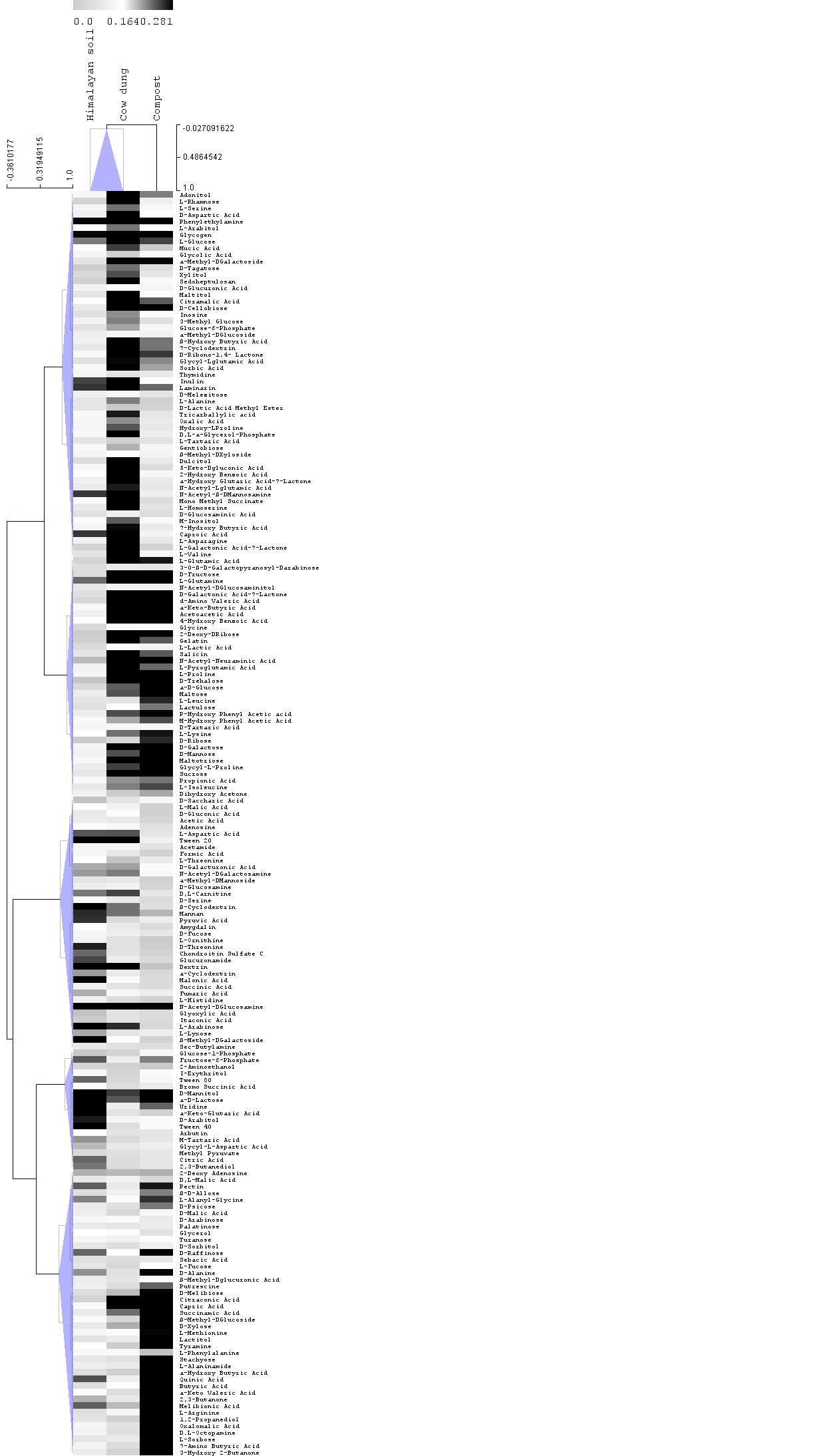


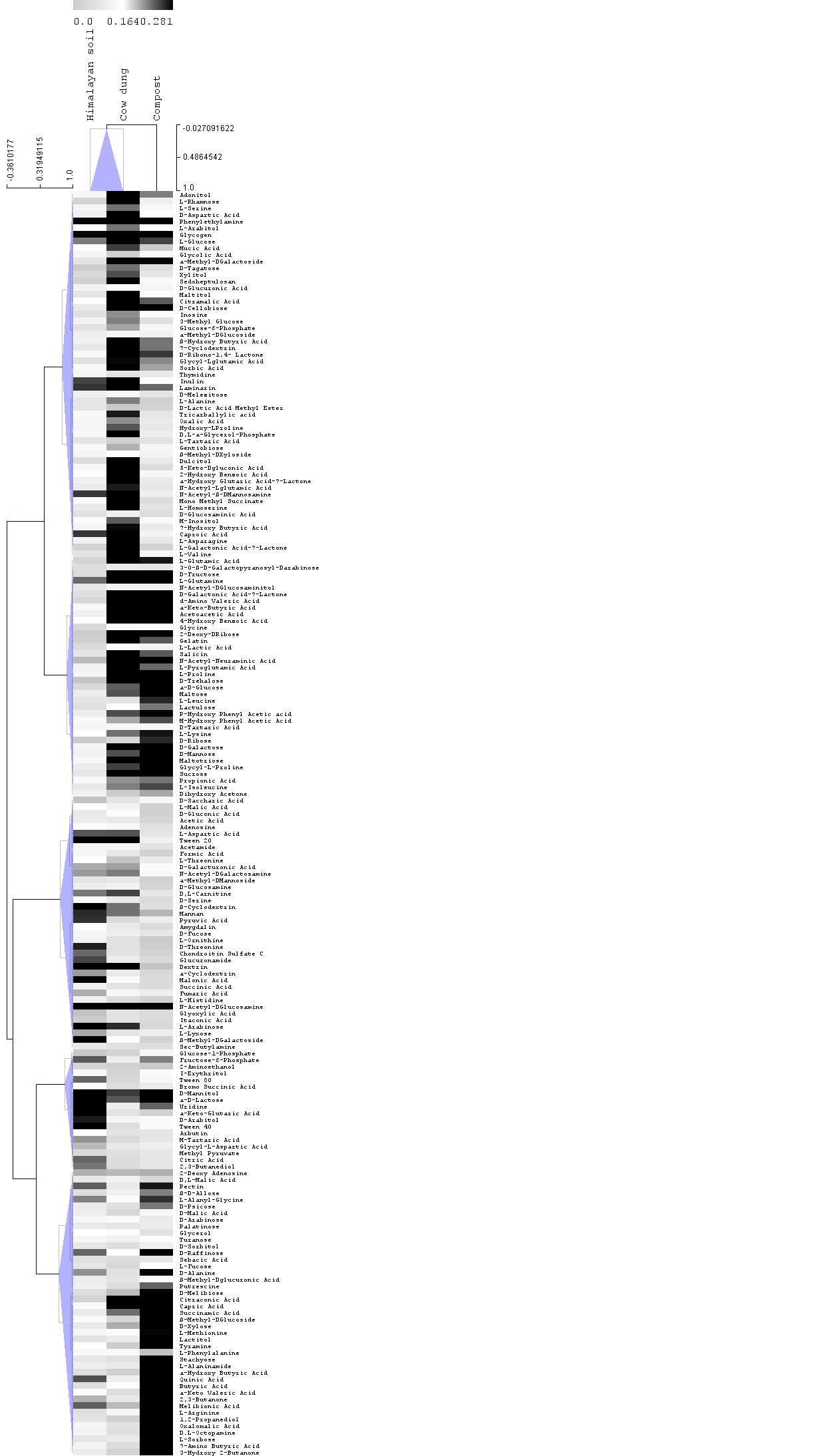


**[B]**


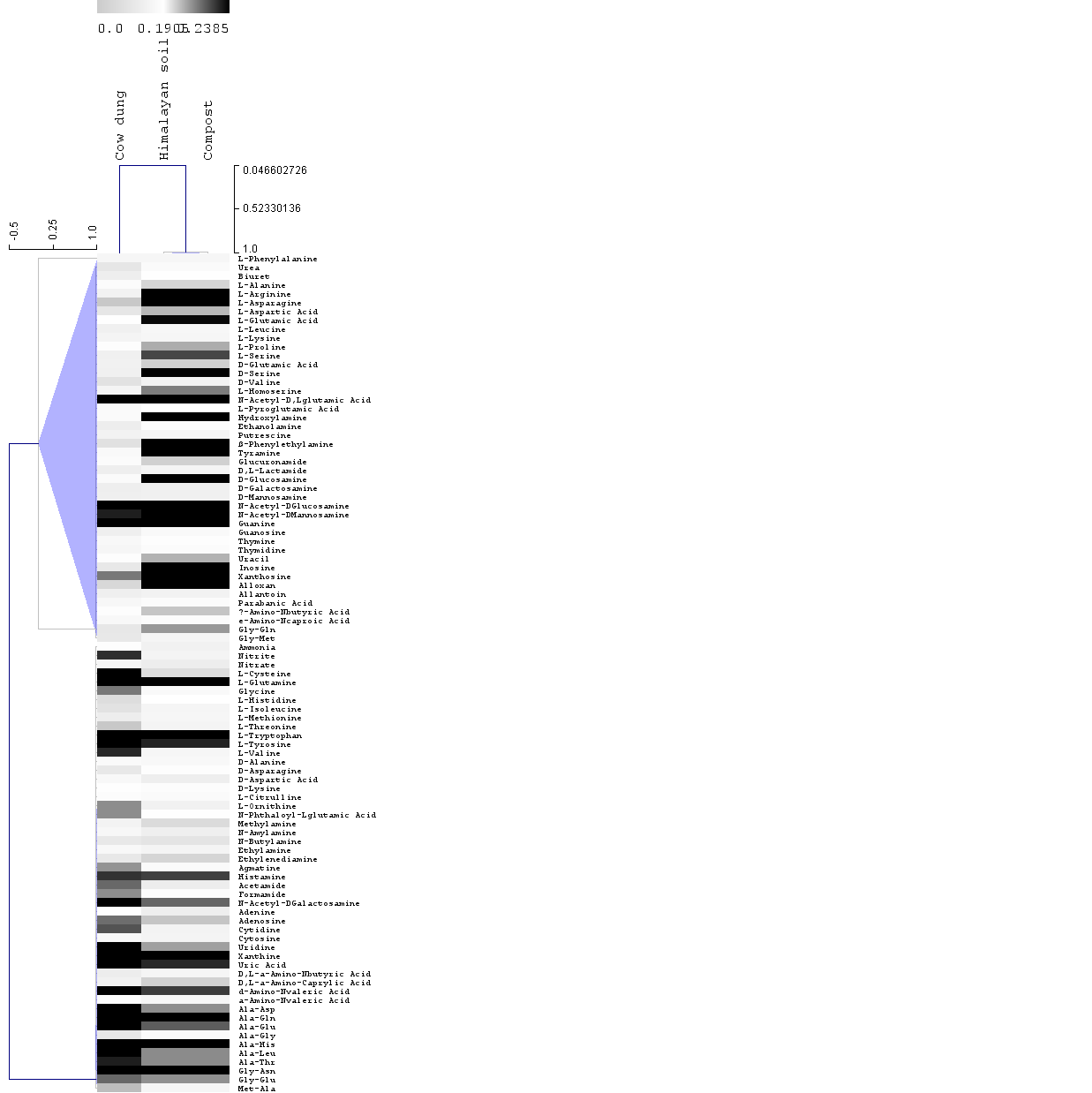


**[C]**


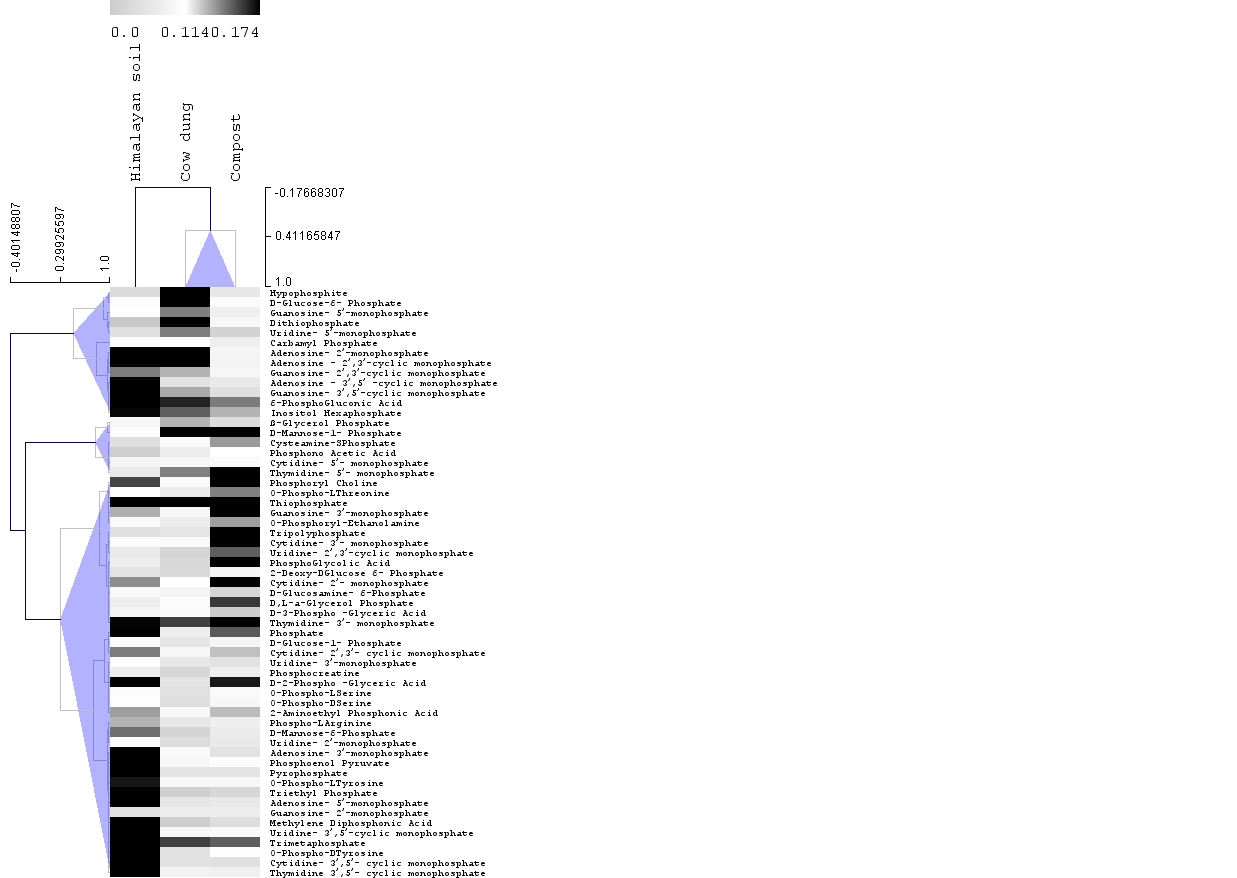


**[D]**


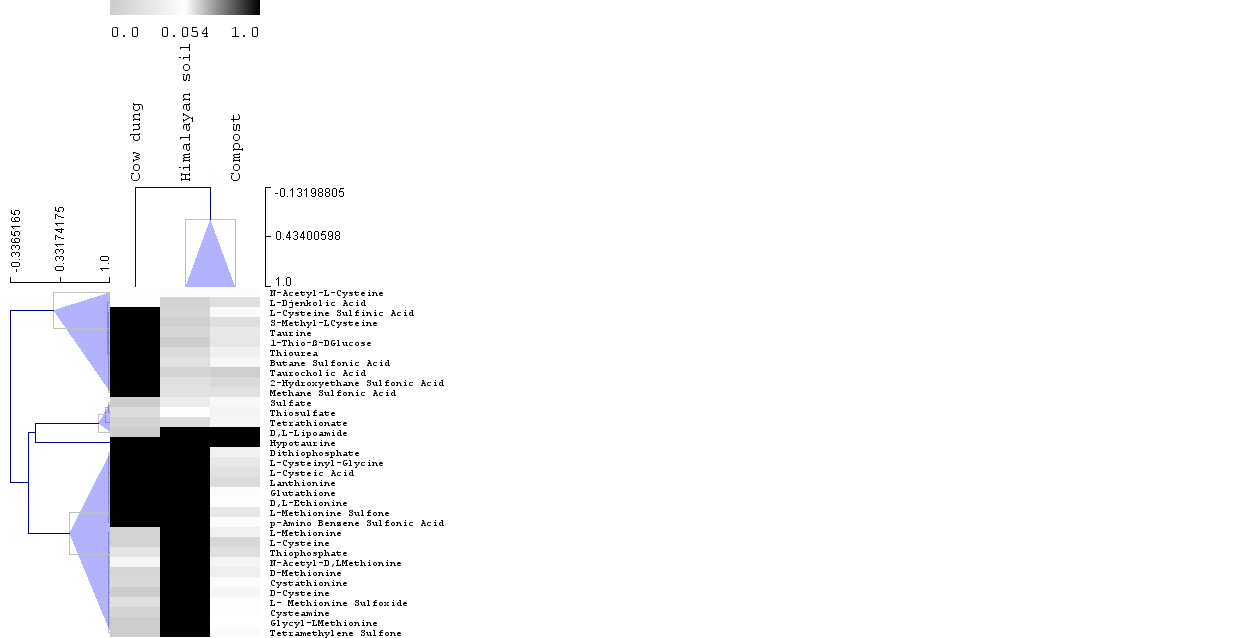


**Supplementary Figure** **1.** The cluster heatmap generated using Multiple array viewer (MeV 4.9.0) software package with distance node as the similarity measure and hierarchical clustering with complete linkage of carbon [A], nitrogen [B], phosphorus [C] and sulphur [D] substrates.

**
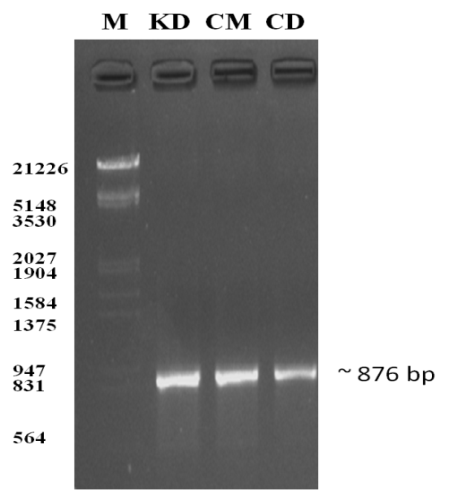
**

**Supplementary Figure 2.** PCR amplification of partial β-glucosidase (BGL) gene and 876 bp fragment from metagenomic DNA of Himalayan region, Kargil district (KD), India, Cow dung (CD) and Compost (CM). lane M, Lambda DNA/*Eco*RI+*Hind*III Marker.


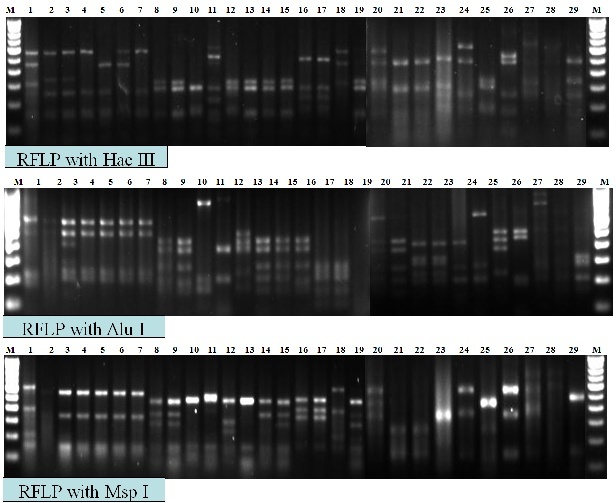

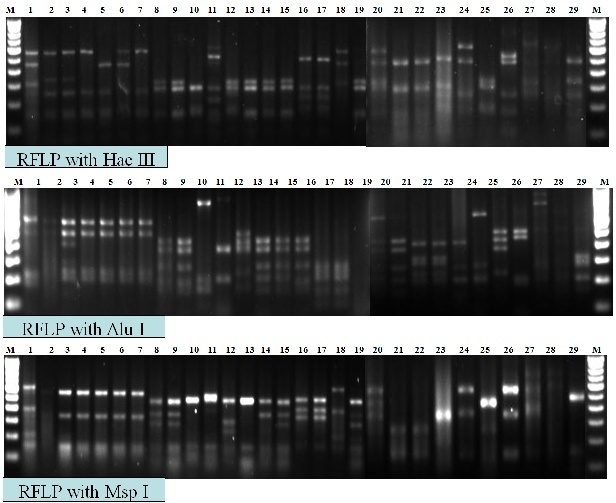

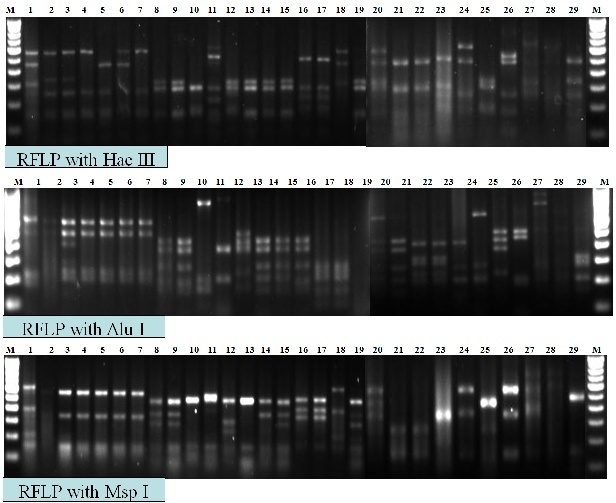


**Supplementary Figure 3.** RFLP fingerprinting patterns of partial β-glucosidase (BGL) gene selected from three clonal libraries.
